# Slit/Robo signaling regulates Leydig cell steroidogenesis

**DOI:** 10.1186/s12964-020-00696-6

**Published:** 2021-01-21

**Authors:** Emmanuelle Martinot, Derek Boerboom

**Affiliations:** Département de Biomédecine Vétérinaire, Centre de Recherche en Reproduction Et Fertilité, Faculté de Médecine Vétérinaire, Université de Montréal, Saint-Hyacinthe, QC Canada

**Keywords:** Slit, Robo, Leydig cell, Steroidogenesis, CREB, AKT, LH responsiveness

## Abstract

**Background:**

First identified as a regulator of neuronal axon guidance, Slit/Robo signaling has since been implicated in additional physiologic and pathologic processes, such as angiogenesis, organogenesis and cancer progression. However, its roles in the regulation of testis function have been little explored.

**Methods:**

Immunohistochemistry and RT-qPCR analyses were performed to detect the expression of Slit/Robo signaling effectors in the adult mouse testis. To identify the roles and mechanisms of Slit/Robo signaling in the regulation of steroidogenesis, RT-qPCR, immunoblotting and hormone measurements were carried out using Leydig cells (primary cultures and the MA10 cell line) treated with exogenous SLIT ligands, and testes from *Robo1*-null mice.

**Results:**

S*lit1, -2* and *-3* and *Robo1* and *-2* expression was detected in the adult mouse testis, particularly in Leydig cells. In vitro treatment of Leydig cells with exogenous SLIT ligands led to a decrease in the expression of the steroidogenic genes *Star*, *Cyp11a1*, and *Cyp17a1*. SLIT2 treatment decreased the phosphorylation of the key steroidogenic gene regulator CREB, possibly in part by suppressing AKT activity. Furthermore, SLIT2 treatment reduced the responsiveness of MA10 cells to luteinizing hormone by decreasing the expression of *Lhcgr*. Consistent with these in vitro results, an increase in testicular *Star* mRNA levels and intra-testicular testosterone concentrations were found in *Robo1*-null mice. Finally, we showed that the expression of the *Slit* and *Robo* genes in Leydig cells is enhanced by testosterone treatment in vitro, by an AR-independent mechanism.

**Conclusion:**

Taken together, these results suggest that Slit/Robo signaling represents a novel mechanism that regulates Leydig cell steroidogenesis. It may act in an autocrine/paracrine manner to mediate negative feedback by testosterone on its own synthesis.

**Video Abstract**

## Background

The testis has two main functions: the production of spermatozoa and the synthesis of steroid hormones, mainly androgens. The latter are responsible for the masculinization of the genital tract during fetal development, the development of secondary sexual characteristics at puberty, and the initiation and maintenance of spermatogenesis during adulthood [1, 2]. Testicular steroidogenesis is ensured by the Leydig cells, and is mainly under the control of luteinizing hormone (LH) secreted by pituitary gonadotrope cells in response to gonadotropin-releasing hormone (GnRH). LH interacts with its membrane receptor, LHCGR, to activate Protein Kinase A (PKA) via the production of cAMP by adenylate cyclase [3]. Once activated, PKA phosphorylates the transcription factor cAMP-response element binding protein (CREB) on Ser133, promoting the recruitment of co-activators and the transcription of steroidogenic genes [4]. Besides PKA, CREB can also be phosphorylated at Ser133 by other receptor-activated protein kinases such as calmodulin-dependent protein kinase (CaMK), mitogen-activated protein kinases (MAPK), and thymoma viral proto-oncogene (AKT) [5–7]. Beyond LH, testosterone itself can regulate its own synthesis, on the one hand via negative feedback on the synthesis and secretion of LH, and on the other hand by acting on Leydig cells in an autocrine/paracrine manner [8–10].

SLIT ligands are secreted glycoproteins which bind cognate single-pass transmembrane receptors of the roundabout (ROBO) family [11, 12]. In vertebrates, 3 *SLIT* (*1–3*) and 4 *ROBO* genes (*1–4*) have been characterized [13, 14]. Whereas ROBO1 and ROBO2 are structurally and functionally similar, mammalian ROBO3 is structurally distinct and lacks the ability to bind SLIT ligands [15]. *ROBO4*, which is primarily expressed in vascular endothelial cells, encodes an even more highly divergent receptor with shorter extracellular and cytoplasmic regions, and its capacity to directly interact with SLIT ligands is still debated [15, 16]. First identified as important regulators of axon guidance during nervous system development [17], Slit/Robo family members have since been found to be involved in the regulation of cell adhesion, proliferation and survival in a wide variety of tissues, thereby participating in angiogenesis, organogenesis and tumor progression [18, 19]. As ROBO receptors lack any enzymatic or autocatalytic activity, SLIT/ROBO complexes mediate their functions via the recruitment of cytoplasmic signaling and scaffolding molecules, including several kinases and regulatory molecules that modify actin and the microtubule cytoskeleton. Notably, ROBO receptors can interact with Slit/Robo GTPase activating proteins (srGAPs) which regulate the activity of Rho GTPases (Cdc42, RhoA, Rac), which in turn alter cell polarity and mobility by modulating the cytoskeleton in neurons and other cell types [20]. Furthermore, *Slit* and *Robo* are considered tumor suppressor genes, as they are frequently inactivated in various tumors. For instance, in breast and lung cancer cells, *Slit2* expression is frequently lost, and overexpression of *Slit2* was associated with an increase in cell adhesion and a decrease in cell proliferation [21]. In this context, SLIT2 appears to signal via AKT/GSK3β, thereby modulating the stability and the transcriptional regulatory activity of the proto-oncogene CTNNB1 (β-catenin) [22, 23].

Slit/Robo signaling also appears to have important functions in gonadal physiology. In the ovary, Slit/Robo family members are expressed in human and mouse corpora lutea, and blocking Slit/Robo signaling leads to a significant decrease in apoptosis in cultured luteal cells [24, 25]. Female mice heterozygous-null for both *Robo1* and *Robo2* were found to be hyperfertile, with a decrease in granulosa cell apoptosis and follicle atresia [26]. Furthermore, manipulating *Slit2* expression in granulosa cells from hen prehierarchical follicles was found to alter *Star* and *Cyp11a1* mRNA expression, suggesting that *Slit/Robo* signaling could also modulate ovarian steroidogenesis [27]. In contrast with the ovary, very little is known about Slit/Robo signaling in the testes. Only two studies to date in drosophila showed that /Robo pathway participates in gonad formation, by contributing to somatic gonadal precursor cluster formation and gonad compaction, as well as by controling the ability of somatic cyst stem cells to compete for occupancy in the testis niche [28, 29].

In the present study, we sought to determine the potential role(s) of Slit/Robo signaling in the mammalian testis. We determined that Slit/Robo signaling, potentially via AKT, is a potent inhibitor of Leydig cell steroidogenesis. As the expression of the SLIT ligands was found to be up-regulated by testosterone, Slit/Robo may therefore represent a novel mechanism whereby testosterone regulates its own biosynthesis.

## Methods

### Animals

C57Bl/6J mice for Leydig cell primary culture were obtained from the Jackson Laboratory (Bar Harbor, ME). *Robo1*-null mice were obtained from Dr Alain Chédotal (Sorbonne Université, INSERM, CNRS, Institut de la Vision, Paris, France), and genotype analyses were done as previously described [30]. Animals were housed in temperature-controlled rooms with 14/10 h light/dark cycles, and had ad libitum access to food and water. All animal procedures were approved by the Institutional Animal Care and Use Committee and conformed to the International Guiding Principles for Biomedical Research Involving Animals.

### In vivo experiment

Two to four month-old wild type mice received an i.p. injection of vehicle (sesame oil) or 1 mg of testosterone (MilliporeSigma, Oakville, ON, Canada) 24 h prior to euthanasia.

### MA10 and MLTC1 cell culture

MA10 and MLTC1 cells were cultured in DMEM/F-12 medium (Thermo Fisher Scientific, Waltham, MA, USA) supplemented with Penicillin/Streptomycin (Wisent, Saint-Jean-Baptiste, QC, Canada) and 15% horse serum (Thermo Fisher Scientific), and in DMEM medium (Thermo Fisher Scientific) supplemented with 10 mM Hepes (BioShop, Burlington, ON, Canada), 40 mM NaHCO3 (BioShop), Penicillin/Streptomycin and 10% fetal bovine serum (MilliporeSigma), respectively, at 37 °C in an atmosphere containing 5% CO_2_. Cells were seeded respectively at 75 × 10^3^ or 60 × 10^3^ cells per well in 48-well plates. The next day, the culture medium was replaced with a serum-free medium, and the cells incubated overnight before treatment with vehicle (PBS) or SLIT recombinant mouse protein (R&D Systems, Minneapolis, MN, USA, SLIT1 #5199-SL, SLIT2 #5444-SL, SLIT3 #9295-SL) at varying concentrations and for varying times. In some experiments, cells were then treated with human Luteinizing Hormone (50 ng/mL, National Hormone and Peptide Program, Los Angeles Biomedical Research Institute, Los Angeles, CA, USA), Forskolin (10 µM, Selleckchem, Burlington, ON, Canada), or the corresponding vehicle (PBS and DMSO, respectively) for 4 h. Alternatively, cells were treated with vehicle (DMSO), wortmannin (100 nM, TOCRIS, Oakville, ON, Canada), or SC79 (20 µg/ml, MilliporeSigma) for 1 h and 4 h. Cells were also treated with vehicle (ethanol), or 5α-dihydro-11-keto testosterone (1 nM, 2 h, Cayman Chemical, Ann Arbor, MI, USA) with or without a pre-treatment with flutamide (20 µM, 1 h, MilliporeSigma).

### Leydig cell primary culture

Leydig cells were isolated from testes of 2- to 4 month-old mice. Testes were decapsulated and digested for 20–30 min at 33 °C in culture medium (DMEM/F-12 with transferrin (5 μg/mL, MilliporeSigma), insulin (5 μg/mL, MilliporeSigma) vitamin E (0.2 μg/mL, MilliporeSigma), EGF (10 ng/mL, MilliporeSigma), glutamine (2.5 mM, Wisent), Penicillin/Streptomycin and 0.1% Fetal Bovine Serum) supplemented with 0.2 mg/mL collagenase (MilliporeSigma), 10 µg/mL DNase I (Roche, Laval, QC, Canada) and 2% Fetal Bovine Serum. Cells were then pelleted by centrifugation (10 min at 200 g and 4 °C) and resuspended in fresh culture medium. After two sedimentation steps, the supernatant containing Leydig cells was centrifuged (10 min at 200 g and 4 °C), and the pellet was resuspended in fresh culture medium at a final concentration of 20–30 × 10^6^ cells per mL. 5 mL of this suspension was deposited on top of a discontinuous Percoll gradient (four layers from 21 to 60%) prepared from a 90% Percoll stock solution (Percoll (GE Healthcare Life Sciences, Marlborough, MA, USA), Ham’s F10 (10X, MilliporeSigma), 20 mM Hepes, 140 mM NaHCO3, pH 7.4) diluted with DMEM/F12 culture medium. Purified Leydig cells were collected at the interface between the 60% and 34% layers after centrifugation (30 min at 500 g and 4 °C), and resuspended in fresh culture medium. Cells were seeded at 45 × 10^4^ cells per well in culture medium in fetal bovine serum-pretreated 48-well plates. The next day, cells were serum starved overnight before treatment with vehicle (PBS) or SLIT2 recombinant mouse protein (10 µg/mL for 8 h).

### Real-time RT-qPCR

Total RNA from whole testis or Leydig cells (MA10 and MLTC1 cell lines or primary culture) was extracted using the RNeasy Mini Kit (Qiagen, Montreal, QC, Canada) according to the manufacturer’s instructions. cDNA was synthesized from 200 ng of RNA using the SuperScript VILO cDNA Synthesis Kit (Thermo Fisher Scientific, Waltham, MA, USA). Real-time PCR reactions were run on a CFX96 Touch Real-Time PCR Detection System (Bio-Rad) using Supergreen Advanced qPCR MasterMix (Wisent). Each PCR reaction consisted of 7.5 μL of Supergreen Advanced qPCR MasterMix, 2.3 μL of water, 4 μL of cDNA sample and 0.6 μL (10 pmol) of gene-specific primers listed in Table 1. DNA fragment amplification was done using the following thermal cycling program: 3 min at 95 °C, followed by 40 cycles of 45 s at 95 °C, 30 s at 60 °C, and 30 s at 72 °C. Relative mRNA levels were determined with the ΔΔCt method using *Rplp0* or *Actb* as the housekeeping gene.

**Table 1:** Quantitative RT-qPCR primer sequences

Gene	Forward	Reverse
*Actb*	TCATCACTATTGGCAACGAGC	AGTTTCATGGATGCCACAGG
*Rplp0*	AGATTCGGGATATGCTGTTGG	AAAGCCTGGAAGAAGGAGGTC
*Cyp11a1*	GTGACCTTGCAGAGGTACACTGT	GTGACTCCAGCCTTCAGTTCACA
*Cyp17a1*	CCAGGACCCAAGTGTGTTCT	CCTGATACGAAGCACTTCTCG
*Hsd3b1*	AGCTGCAGACAAAGACCAAGGTGA	GAACACAGGCCTCCAATAGGTTCT
*Lhcgr*	AGATGCACAGTGGCACCTTCCAG	ATGACGTGGCGATGAGCGTCT
*Slit1*	CTGCTCCCCGGATATGAACC	TAGCATGCACTCACACCTGG
*Slit2*	AACTTGTACTGCGACTGCCA	TCCTCATCACTGCAGACAAACT
*Slit3*	AGTTGTCTGCCTTCCGACAG	TTTCCATGGAGGGTCAGCAC
*Star*	TGTCAAGGAGATCAAGGTCCTG	CGATAGGACCTGGTTGATGAT
*Robo1*	GCTGGCGACATGGGATCATA	AATGTGGCGGCTCTTGAACT
*Robo2*	CTTTGAACGACCCACATTTCTCA	TCTCAGCGTGTAGTCATCTTTGA

### Immunoblotting

MA10 cells were lysed in SDS loading buffer. Protein extracts were resolved on 10% SDS–polyacrylamide gels, and electrophoretically transferred onto Immobilon-P PVDF membranes (MilliporeSigma). Membranes were probed overnight at 4 °C with primary antibodies (Table 2) diluted in Tris-buffered saline with 0.1% tween 20 (TBS-T) containing 5% bovine serum albumin (BSA) (Jackson Laboratory) or 5% dried milk, or for 1 h at room temperature with the anti-ACTB antibody diluted in TBS-T with 5% dried milk. Following incubation with anti-rabbit IgG HRP Conjugate (1:10,000, WB401B, Promega, Madison, WI, USA), signal was generated using Immobilon Western chemiluminescent horseradish peroxidase substrate (MilliporeSigma). Images were captured with the ChemiDoc MP Imaging System (Bio-Rad), and quantified with Image Lab Software v.5.0 (Bio-Rad).

**Table 2:** Antibodies

Antigen	Company	Catalog number	Dilution factor
Antibodies for Western blot analysis			
ACTB	Santa Cruz	47,778	1:10,000
AKT	Cell signaling	4691	1:1500
P-AKT (Ser473)	Cell signaling	4060	1:2000
CREB	Cell signaling	9197	1:1000
P-CREB (Ser133)	Cell signaling	9198	1:1000
GAPDH	Cell signaling	5174	1:10,000
mTOR	Cell signaling	2983	1:1000
P-mTOR (Ser2448)	Cell signaling	2971	1:1000
Antibodies for immunohistochemistry			
ROBO1	Abcam	7279	1:200
SLIT1	Abcam	129,345	1:200
SLIT2	Santa Cruz	514,499	1:50
SLIT3	R&D Systems	MAB3629	1:100

### Immunohistochemistry

Testes and brain cortex were fixed in Bouin's and formaldehyde solution, respectively, embedded in paraffin and sectioned at a thickness of 3 µM. Immunohistochemistry was done using the VECTASTAIN Elite ABC HRP Kit (Vector Laboratories, Burlingame, CA, USA) according to the manufacturer’s instructions. Slides were then stained with the 3,3′-diaminobenzidine peroxidase substrate kit (Vector Laboratories), and counterstained with hematoxylin before mounting. The primary antibodies used are listed in Table 2.

### Hormone measurement

Progesterone (MA10 cell culture medium) and testosterone (Leydig primary cell culture medium and homogenates of testes from 2 and 4 month-old mice) concentrations were determined by ELISA. Serum LH levels were measured by radioimmunoassay. All assays were performed by the Ligand Assay and Analysis Core at the University of Virginia (Charlottesville, VA, USA).

### Statistical analyses

The number of samples per group or replicates per experiment is detailed in the corresponding figure legend. Data are presented as means ± sem. Statistical significance was determined using Student's *t*-test, with *P* ≤ 0.05 considered significant.

## Results

### *Slit* and *Robo* genes are expressed in the Leydig cells of the mouse testis

**Fig. 1:**
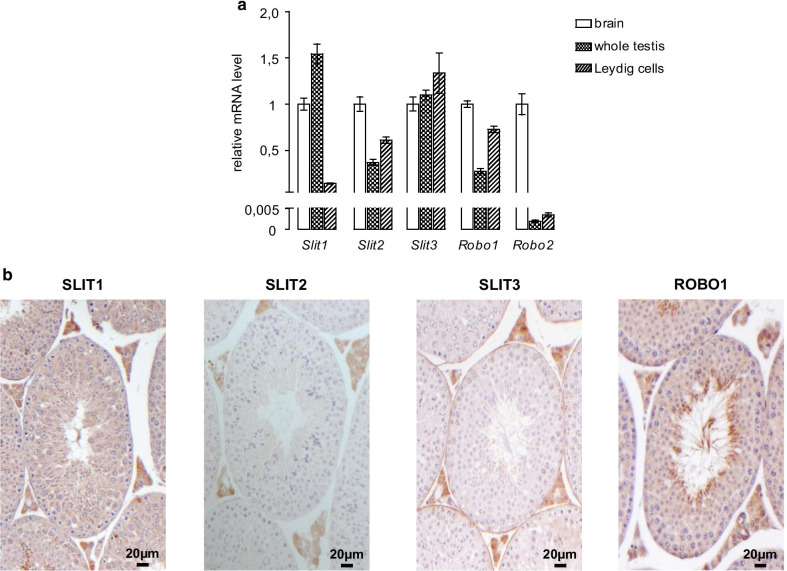
*Slit* and *Robo* genes are expressed in the Leydig cells of the mouse testis. **a** RT-qPCR analyses of brain, testicular and purified Leydig cell *Slit* and *Robo* mRNA levels (n = 4–6 per group). The relative levels are represented as delta-Ct versus *Actb*. Data are means ± sem. **b** SLIT1, -2 and -3 and ROBO1 immunohistochemistry analyses of 8 week-old wild-type mouse testis

We first determined which *Slit* ligands and *Robo* receptors are expressed in the adult mouse testis by RT-qPCR, using a brain sample as a positive control. Transcripts from all three *Slit* genes and the canonical receptors *Robo1* and *Robo2* were detected, although *Robo2* was expressed at very low level (Fig. 1a). We then used immunohistochemistry to examine the location of *Slit/Robo* expression within the testis. ROBO1, SLIT1, -2 and -3 were all found to be strongly expressed, specifically in the interstitial compartment (Fig. 1b), with low levels of SLIT1 and ROBO1 also detectable within the seminiferous tubules. The specificity of the staining was validated using negative (no primary antibody) and positive (brain cortex) controls (Additional file 1: Fig. S1). These results were confirmed at the mRNA level, as all aforementioned *Slit/Robo* family members were detected in a Leydig cell-enriched sample (Fig. 1a). Although mRNA levels of *Robo1* and *-2* and *Slit2* and *-3* were higher in Leydig cells compared to the whole testis, the opposite was observed for *Slit1*. Together with the immunohistochemistry analysis, this suggests that *Slit2* and *-3* are predominantly expressed in Leydig cells, whereas *Slit1* may be predominantly expressed in other testicular cell types.

### Exogenous SLIT ligands decrease steroidogenesis in Leydig cells in vitro

**Fig. 2:**
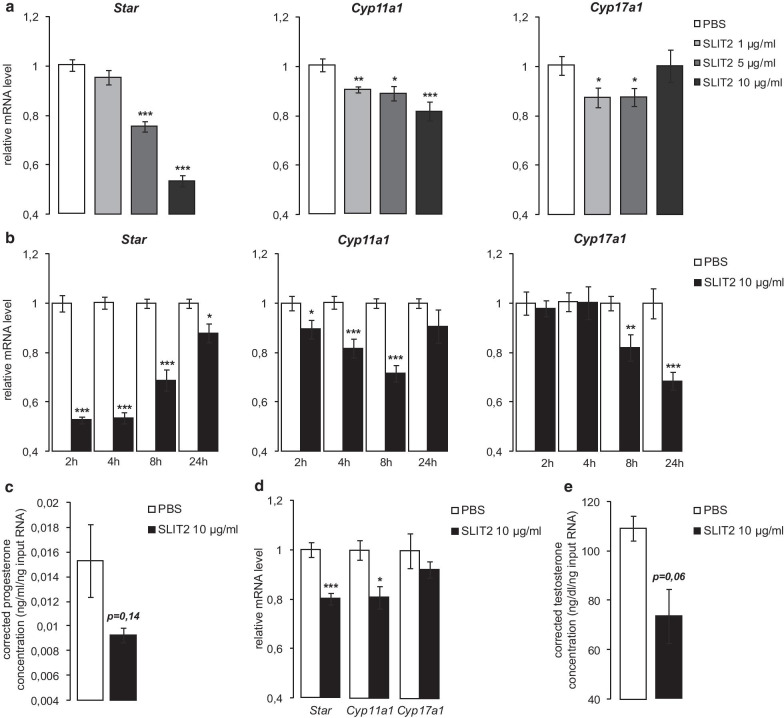
Exogenous SLIT2 decreases steroidogenesis in Leydig cells in vitro. Expression of *Star*, *Cyp11a1* and *Cyp17a1* determined by RT-qPCR in MA10 cells treated **a** for 4 h with vehicle or 1, 5 and 10 µg/ml exogenous SLIT2; **b** for 2, 4, 8 and 24 h with vehicle or 10 µg/ml SLIT2. **c** Representative graph of progesterone concentrations (corrected to RNA input) measured in the spent culture media of MA10 cells treated for 8 h with vehicle or 10 µg/ml SLIT2. **d** Expression of *Star*, *Cyp11a1* and *Cyp17a1* determined by RT-qPCR and **e** representative graph of testosterone concentrations (corrected to RNA input) measured in the spent culture media of mouse primary Leydig cell cultures treated for 8 h with vehicle or 10 µg/ml SLIT2. Experiments were performed three times in triplicate. Expression of each transcript was normalized to the housekeeping gene *Rplp0*. Data are means ± sem; statistical analysis (Student’s *T*-test): *p < 0.05; **p < 0.01; ***p < 0.001

To determine the role of Slit/Robo signaling in Leydig cells, MA10 cells were treated with graded concentrations of exogenous SLIT2 for 4 h, and the mRNA levels of genes encoding steroidogenic enzymes were measured. The addition of SLIT2 decreased *Star* and *Cyp11a1* mRNA levels in a dose-dependent manner (Fig. 2a). *Cyp17a1* expression was also significantly decreased, but only at the 1 and 5 µg/mL concentrations (Fig. 2a). *Hsd3b1* mRNA levels were not altered by SLIT2 treatment (not shown). A time course analysis was then done using SLIT2 at 10 µg/mL. A decrease in *Star* and *Cyp11a1* mRNA levels was observed at all time points examined, although in both cases this effect had begun to reverse itself after 24 h of treatment (Fig. 2b). *Cyp17a1* mRNA levels also decreased in response to SLIT2, but more gradually than *Star* or *Cyp11a1*, with the decrease becoming statistically significant by 8 h (Fig. 2b). Progesterone concentrations in spent culture medium also appeared lower 8 h following addition of SLIT2, although the effect was not statistically significant (Fig. 2c). A similar inhibitory effect on steroidogenic gene expression was observed when MA10 cells were treated with exogenous SLIT1, but not with exogenous SLIT3 (Additional file 1: Fig. S2A). However, in another Leydig cell line (MLTC1), all three SLIT recombinant proteins were able to down-regulate the expression of the steroidogenic genes (Additional file 1: Fig. S2B), suggesting they may act in a functionally redundant manner. Findings obtained in cell lines were then confirmed in primary cultured Leydig cells. The efficiency of Leydig cell isolation was validated by RT-qPCR using specific Leydig, Sertoli and germ cell markers (*Star*, *Fshr* and *Dmc1*, respectively). As expected, *Star* was enriched whereas *Fshr* and *Dmc1* were expressed at very low level in the isolated Leydig cells compared to whole testis samples (Additional file 1: Fig. S3). In these cells, we observed a significant decrease of *Star* and *Cyp11a1* mRNA levels in response to SLIT2, with testosterone concentrations trending lower (*P* = 0.06) 8 h after treatment (Fig. 2d, e). Together, these results suggest that SLIT ligands can antagonize Leydig cell steroidogenesis by regulating steroidogenic gene expression in a time- and dose-dependent manner.

### Exogenous SLIT2 decreases CREB phosphorylation

**Fig. 3:**
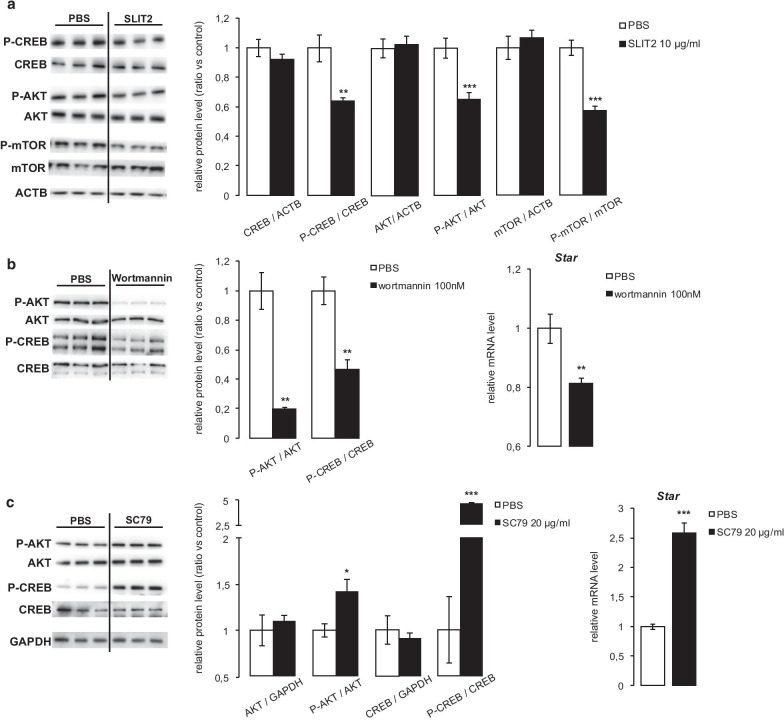
Exogenous SLIT2 decreases CREB phosphorylation. **a** Quantification of total and phosphorylated CREB, AKT and mTOR protein levels normalized to ACTB in MA10 cells treated for 1 h with vehicle or 10 µg/ml SLIT2. Quantification of total and phosphorylated AKT and CREB protein levels normalized to GAPDH, and expression of *Star* determined by RT-qPCR, in MA10 cells treated with vehicle or **b** 100 nM wortmannin, or **c** 20 µg/ml SC79, for 1 h (protein) or 4 h (mRNA). n = 3–9 samples per group. Expression of *Star* was normalized to the housekeeping gene *Rplp0*. Data are means ± sem; statistical analysis (Student’s *T*-test): *p < 0.05; **p < 0.01; ***p < 0.001

CREB is one of the most important transcription factors involved in the regulation of the expression of steroidogenic genes, and is activated by phosphorylation. Interestingly, treating MA10 cells for 1 h with SLIT2 resulted in a significant decrease in CREB (Ser133) phosphorylation (Fig. 3a).

AKT is a Ser/Thr kinase able to phosphorylate CREB at Ser133 [7]. Importantly, its phosphorylation and activity can be inhibited by Slit/Robo signaling [22], leading us to hypothesize that SLIT2 may suppress steroidogenesis via an AKT-dependent mechanism. We therefore examined the effects of SLIT2 on AKT phosphorylation and activity. AKT phosphorylation decreased significantly in response to SLIT2 (Fig. 3a), and this was correlated with a decrease in phosphorylation of its substrate mTOR (Fig. 3a). Interestingly, the PI3K/AKT inhibitor wortmannin reproduced the decrease in CREB phosphorylation and *Star* mRNA levels observed in response to SLIT2 (Fig. 3b), whereas the opposite effect was observed with the AKT activator SC79 (Fig. 3c). The efficacy of these 2 drugs was validated as they respectively generated a decrease and an increase in AKT phosphorylation (Fig. 3b, c). AKT is therefore able to regulate CREB and Leydig cell steroidogenesis, and its activity can be regulated by Slit/Robo signaling.

### Exogenous SLIT2 alters Leydig cell responsiveness to LH by decreasing *Lhcgr* expression

**Fig. 4:**
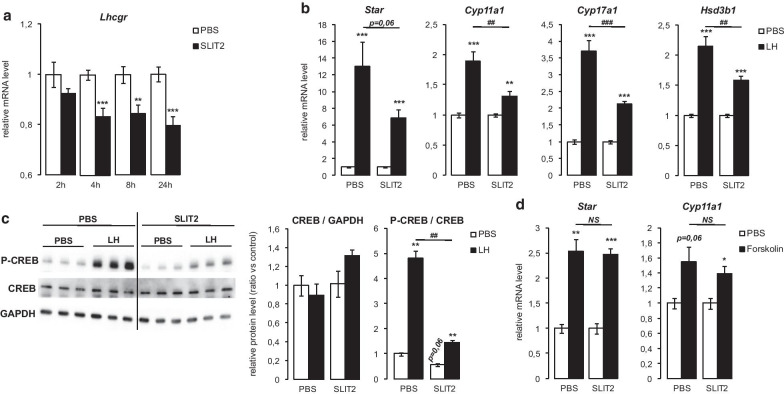
Exogenous SLIT2 alters Leydig cell responsiveness to LH by decreasing *Lhcgr* expression. **a** Expression of *Lhcgr* determined by RT-qPCR in MA10 cells treated for 2, 4, 8 and 24 h with vehicle or 10 µg/ml SLIT2. **b** Expression of *Star*, *Cyp11a1*, *Cyp17a1* and *Hsd3b1* determined by RT-qPCR in MA10 cells treated with 10 µg/ml SLIT2 for 24 h, ± 50 ng/ml LH for 4 h. **c** Quantification of total and phospho-CREB protein levels normalized to GAPDH in MA10 cells treated with 10 µg/ml SLIT2 for 24 h, ± 50 ng/ml LH for 30 min. **d** Expression of *Star* and *Cyp11a1* determined by RT-qPCR in MA10 cells treated with 10 µg/ml SLIT2 for 24 h, ± 10 µM forskolin for 4 h. n = 3–9 samples per group. Expression of each transcript was normalized to the housekeeping gene *Rplp0*. Data are means ± sem; statistical analysis (Student’s *T*-test): *p < 0.05; **p < 0.01; ***p < 0.001; #*p* < 0.05; ##*p* < 0.01; ###*p* < 0.001; *NS* not significant

A significant, time-dependent decrease in *Lhcgr* mRNA levels was observed in MA10 cells in response to SLIT2 treatment (Fig. 4a), suggesting that SLIT2 could alter Leydig cell responsiveness to LH, the most important endocrine regulator of testicular steroidogenesis. To test this, MA10 cells were pre-treated (or not) with exogenous SLIT2 for 24 h prior to stimulation with LH. The increases in the mRNA levels of the steroidogenic genes *Star*, *Cyp11a1*, *Cyp17a1* and *Hsd3b1* (Fig. 4b) and in CREB phosphorylation (Fig. 4c) in response to LH were all blunted in cells pre-treated with SLIT2. However, when the experiment was repeated using forskolin (an activator of adenylate cyclase) in lieu of LH, differences in forskolin-stimulated steroidogenic gene mRNA levels between cells pre-treated with vehicle or SLIT2 were not observed (Fig. 4d). The antagonism of LH-stimulated steroidogenic gene expression by SLIT2 therefore seems to be mediated, at least in part, by its suppression of *Lhcgr* expression.

### Increased testicular steroidogenesis in *Robo1-*null mice

**Fig. 5:**
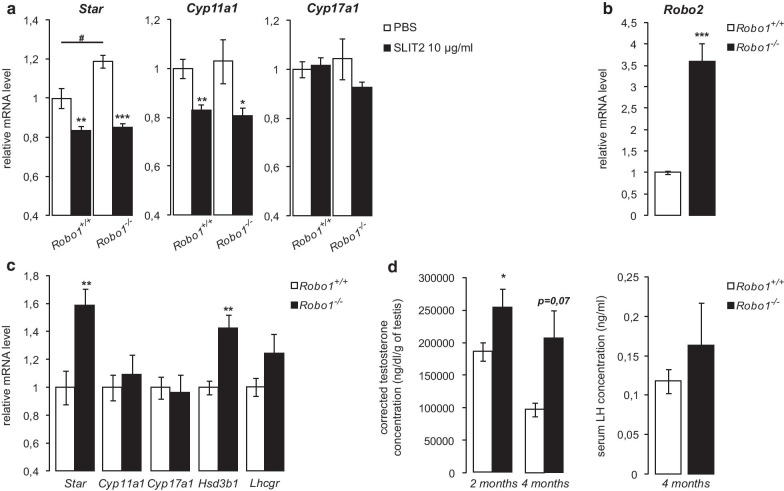
Increased testicular steroidogenesis in *Robo1-*null mice. **a** Expression of *Star*, *Cyp11a1* and *Cyp17a1* determined by RT-qPCR in primary Leydig cells isolated from *Robo1*^+/+^ vs Robo1*Robo1*^−/−^ mice treated for 8 h with vehicle or 10 µg/ml SLIT2. n = 4 samples per group. **b** Expression of *Robo2* determined by RT-qPCR in cultured Leydig cells isolated from *Robo1*^+/+^ and *Robo1*^−/−^ mice. n = 4 samples per group. **c** Expression of *Star*, *Cyp11a1*, *Cyp17a1*, *Hsd3b1* and *Lhcgr* determined by RT-qPCR in testes from four month-old *Robo1*^+/+^ and *Robo1*^−/−^ mice. n = 5–7 per group. Expression of each transcript was normalized to the housekeeping gene *Rplp0*. **d** Intra-testicular testosterone concentrations corrected to testis weight measured in two- and four month-old *Robo1*^+/+^ and *Robo1*^−/−^ mice, and serum LH levels measured in four month-old mice. n = 5–10 per group. Data are means ± sem; statistical analysis (Student’s *T*-test): *p < 0.05; **p < 0.01; ***p < 0.001; #*p* < 0.05; ##*p* < 0.01; ###*p* < 0.001

As *Robo1* appeared to be the predominantly expressed canonical SLIT receptor in Leydig cells (Fig. 1), we sought to determine if ROBO1 mediates the inhibitory effect of SLIT2 on steroidogenesis. Leydig cells isolated from wild-type (*Robo1*^+/+^) and *Robo1*-null (*Robo1*^−/−^) mice were treated with vehicle or exogenous SLIT2 for 8 h. SLIT2 treatment led to a significant decrease in *Star* and *Cyp11a1* expression in Leydig cells from animals of both genotypes (Fig. 5a), indicating that *Robo1* is not essential in this context. RT-qPCR analyses showed 3.5-fold higher *Robo2* mRNA levels in Leydig cells from *Robo1*^−/−^ mice relative to controls (Fig. 5b), suggesting both the existence of a compensatory mechanism and the functional redundancy of *Robo1* and *-2* in mediating SLIT2 action in Leydig cells.

Interestingly, *Star* mRNA levels were significantly higher in untreated primary cultures of Leydig cells from *Robo1*^−/−^ mice (Fig. 5a), consistent with *Star* being the gene most responsive to exogenous SLIT ligands in vitro (Fig. 2). These results prompted us to examine testicular steroidogenesis in *Robo1*^−/−^ mice. Four month-old *Robo1*^−/−^ mice were found to have increased testicular *Star* and *Hsd3b1* mRNA levels relative to their wild-type littermates, whereas no difference was observed for *Cyp11a1* or *Cyp17a1* (Fig. 5c). *Lhcgr* expression also tended to increase in the mutant animals (Fig. 5c). These results were associated with increased intra-testicular testosterone levels in *Robo1*^−/−^ mice at two and four months of age (Fig. 5d). However, no alterations in serum LH levels (Fig. 5d), testis weights, sperm counts, accessory sex organ weights or male fertility were found in *Robo1*^−/−^ mice (not shown).

### The expression of *Slit* and *Robo* genes is regulated by testosterone

**Fig. 6:**
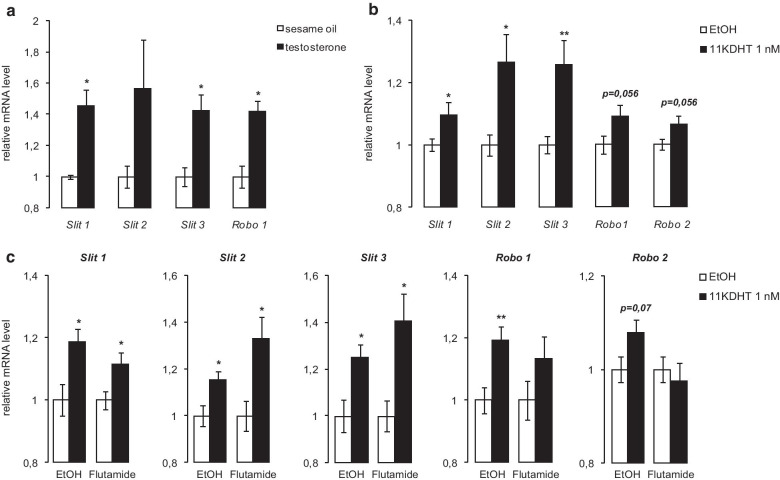
The expression of *Slit* and *Robo* genes is regulated by testosterone. Expression of *Slit* and *Robo* genes determined by RT-qPCR in **a** testes of 2- to 4 month-old wild type mice treated for 24 h with vehicle or 1 mg testosterone (n = 3 per group); **b** MA10 cells treated for 2 h with vehicle or 1 nM 5α-dihydro-11-keto testosterone (performed three times in triplicate), and **c** MA10 cells treated for 2 h with vehicle or 1 nM 5α-dihydro-11-keto testosterone ± 20 µM flutamide for 1 h (performed twice in triplicate). Expression of each transcript was normalized to the housekeeping gene *Actb* or *Rplp0*, respectively. Data are means ± sem; statistical analysis (Student’s *T*-test): *p < 0.05; **p < 0.01; ***p < 0.001

**Fig. 7:**
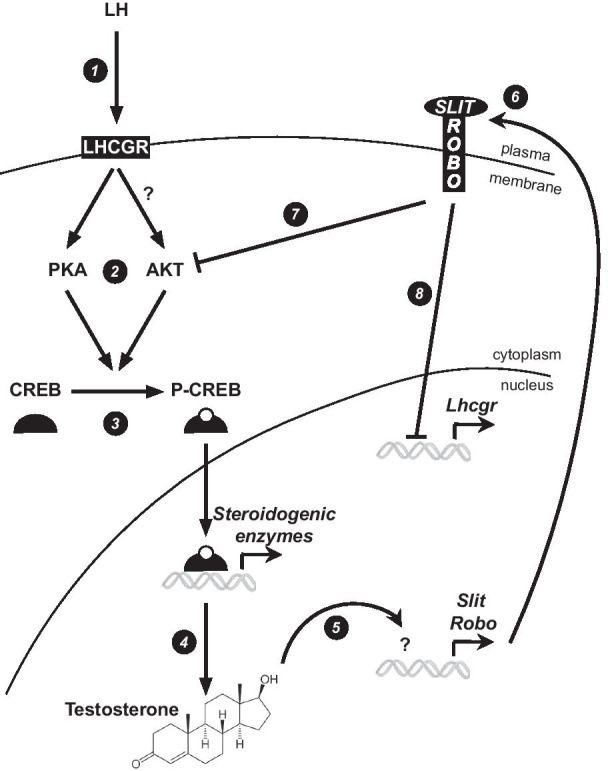
Working model of Slit/Robo feedback regulation of testosterone synthesis. LH interacts with its membrane receptor, LHCGR (1), to promote the phosphorylation of the transcription factor CREB by PKA and (potentially) AKT (2, 3). This leads to the activation of the transcription of steroidogenic genes and testosterone synthesis (4). Testosterone then enhances the expression of *Slit* and *Robo* genes (5, 6) to decrease Leydig cell steroidogenic activity by antagonizing LH signaling. This is potentially achieved both by decreasing AKT-induced CREB phosphorylation (7) and by decreasing Lhcgr expression (8)

Finally, we investigated the physiological context in which Slit/Robo signaling might be activated to inhibit Leydig cell steroidogenesis. As the expression of *Slit*/*Robo* genes has been shown to be modulated by steroids [24, 31], we examined the effect of androgens on their mRNA levels in vivo and in vitro. Injection of 1 mg of testosterone in wild-type mice led to an increase in testicular *Slit1, -2, -3* and *Robo1* mRNA levels, which was significant for all these genes except for *Slit2* (Fig. 6a). In order to determine if androgens regulate *Slit/Robo* genes expression more specifically in Leydig cells, MA10 cells were treated with 5α-dihydro-11-keto testosterone (1 nM) for 2 h. This caused the mRNA levels of all *Slit* genes, *Robo1* and *Robo2* to either trend higher or increase significantly (Fig. 6b), suggesting an autoregulatory feedback loop on Leydig cell steroidogenesis (Fig. 7). To determine if the androgen receptor (AR) is involved in the regulation of *Slit*/*Robo* genes by androgens, the MA10 cell line was pre-treated for 1 h with the AR inhibitor Flutamide prior to addition of 5α-dihydro-11-keto testosterone. 5α-dihydro-11-keto testosterone was able to increase the mRNA levels of *Slit/Robo* genes in absence or in presence of Flutamide (Fig. 6c), suggesting that the mechanism of their regulation by androgens is AR-independent.

## Discussion

Although LH is widely regarded as the major endocrine regulator of testicular steroidogenesis, many studies have now shown that testosterone synthesis by Leydig cells is also modulated by a number of autocrine/paracrine-acting factors. These include growth factors and cytokines such as IGF1, FGF9, TGFβ1, TNFα, AMH, Il1 and Il6 [32]. Importantly, testosterone itself also regulates its own synthesis, as shown by several studies that have employed gene targeting techniques in mice to inactivate the androgen receptor (AR) in Leydig cells [9, 10]. With this study, we propose that Slit/Robo signaling represents a novel autocrine mechanism that regulates Leydig cell steroidogenesis. The negative effect of SLIT treatment of Leydig cells in vitro on *Lhcgr* and steroidogenic gene expression, CREB phosphorylation and LH signaling, coupled with increased Leydig cell *Star* expression and testosterone synthesis in *Robo1*-null mice, all argue that the physiological role of Slit/Robo signaling in Leydig cells is to antagonize LH-driven steroidogenesis.

Whereas the ability of testosterone to regulate its own synthesis is well-established, how it does so remains unknown, other than that AR is a key mediator of its action [9, 10]. Here, we show that testosterone up-regulates the expression of *Slit* and *Robo* genes in Leydig cells, creating a potential negative feedback loop that would permit high local testosterone levels to induce Slit/Robo signaling, thereby inhibiting *Lhcgr* expression and LH signaling, and resulting in decreased testosterone synthesis (Fig. 7). Although further validation of this working model is clearly warranted, this mechanism nonetheless suggests a novel level of local control over steroidogenesis that could fine-tune the endocrine LH signal. In our model, androgens seem to regulate *Slit* and *Robo* gene expression in an AR-independent manner. Such AR-independent actions of androgens have been described in several cell types, including macrophages, T cells and neuroblastoma cells [33–35]. In these cells, testosterone induces a rapid rise in intracellular Ca^2+^ levels that is not affected by pharmacologic inhibitors of AR. Elevated Ca^2+^ in turn induces signal transduction cascades and modulation of transcription [36]. How testosterone regulates *Slit* and *Robo* gene expression in Leydig cells will be grounds for further study, but a Ca^2+^- dependant mechanism could certainly be involved.

Further mechanistic investigation will also be required to better define how Slit/Robo antagonizes LH signaling. In this report, we show that SLIT2 treatment of Leydig cells reduces *Lhcgr* levels, presumably thereby decreasing their responsiveness to LH. Whether the observed decrease in AKT activity that occurs in response to SLIT2 represents the signaling mechanism that drives the decrease in *Lhcgr* expression remains to be determined. As CREB is a substrate of both PKA and AKT, another possibility is that Slit/Robo antagonizes LH signaling by decreasing CREB phosphorylation (Fig. 7). Indeed, our data suggest that both basal and LH-induced CREB phosphorylation are decreased by SLIT2 treatment, indicating that some level of AKT activity must be present for optimal CREB phosphorylation and a robust LH response. The signals that drive AKT activity in Leydig cells in this context will need to be established, but one possibility is LH itself, which signals via AKT in ovarian theca interna cells [37, 38], and may do so in Leydig cells as well [39].

Our descriptive data showed higher expression of *Slit2* and *-3* and *Robo1* in a Leydig cell-enriched sample relative to whole testes, suggesting that they are primarily expressed in this cell type. However, some level of expression (and Slit/Robo signaling activity) in other testicular cell type(s) can't be excluded, and indeed would seem likely. Consistent with this, *Slit1* was found to be more expressed in whole testes relative to Leydig cells. As SLITs are secreted proteins, they could thus participate in the regulation of Leydig cell steroidogenesis in both an autocrine and paracrine manner. Conversely, Slit/Robo signaling could also act in other testicular cell types to play roles in the regulation of other functions, such as spermatogenesis, which has not been explored so far in mammals.

A functional redundancy amongst the SLIT ligands and amongst the ROBO receptors has been previously described in the literature [27, 30]. Although SLIT3 appeared less potent than SLIT1 or -2 in the MA10 cell line, all three ligands were similarly effective in MLTC1 cells, also suggesting that functional redundancy occurs in the context of the regulation of Leydig cell steroidogenesis, at least in vitro. To determine which ligand(s) play(s) a preponderant role in the physiologic context will likely require the conditional targeting of all *Slit* genes, individually and in combination. Likewise, our data showing that SLIT2 is equally effective at suppressing steroidogenic gene expression in Leydig cells from wild-type and *Robo1*^−/−^ mice argue that ROBO1 is not the sole canonical SLIT receptor in this cell type. This was an unexpected outcome given our data showing very low levels of *Robo2* expression in the testis, as ROBO2 is the receptor most structurally and functionally related to ROBO1 [18], and hence the likeliest to be able to offset its loss. However, an apparent compensatory increase in *Robo2* mRNA levels was observed in Leydig cells from *Robo1*^−/−^ mice, perhaps enough to restore adequate Slit/Robo signaling for SLIT2 to able to exert its effects. Importantly, *Star* mRNA levels and testosterone synthesis were increased in the testes of *Robo1*^−/−^ mice, suggesting that ROBO1 does indeed transduce a steroidogenesis-inhibitory signal in vivo, and that *Robo2* overexpression does not completely compensate for the loss of *Robo1*.

## Conclusions

In summary, we have identified Slit/Robo as a novel signaling mechanism that regulates Leydig cell steroidogenesis. The induction of *Slit* and *Robo* gene expression by testosterone, coupled with the suppressive effect of Slit/Robo on LH signaling and steroidogenesis, suggest a negative feedback loop that permits testosterone to regulate its own synthesis. Beyond broadening our understanding of the regulation of testicular steroidogenesis, our findings may provide insight into the pathogenesis and/or treatment of disorders of male reproductive tract development, hypogonadism and infertility.

## Supplementary information

**Additional file 1.** Supplemental Figures.

## Data Availability

All data generated or analyzed during this study are included in this published article [and its supplementary information files].

## Abbreviations

AR: Androgen receptor
CAMK: Calmodulin-dependent protein kinase
CREB: CAMP-response element binding protein
Cyp11a1: Cytochrome P450, family 11, subfamily a, polypeptide 1
Cyp17a1: Cytochrome P450, family 17, subfamily a, polypeptide 1
GnRH: Gonadotropin-Releasing hormone
GTPase: GTP hydrolase
Hsd3b1: Hydroxy-delta-5-steroid dehydrogenase, 3 beta-and steroid delta-isomerase 1
LH: Luteinizing hormone
MAPK: Mitogen-activated protein kinase
PKA: Protein kinase A
srGAP: Slit/Robo GTPase activating proteins
Star: Steroidogenic acute regulatory protein
