# Supplementary material for: Slit/Robo signaling regulates Leydig cell steroidogenesis
Source: Cell Commun Signal. 2021 Jan 21;19:8. doi: 10.1186/s12964-020-00696-6 (PMC7819258; doi:10.1186/s12964-020-00696-6)
Supplement: Supplementary file 2 — Additional file 1. Supplemental Figures. [file 12964_2020_696_MOESM2_ESM.docx]

**Additional file 1: Figure S1. Validation of Slit1, -2 and -3 and Robo1 antibodies.** *IHC negative (no primary antibody) and positive (brain cortex) controls for Slit1, -2 and -3 and Robo1 antibodies.*

**Additional file 1: Figure S2. Exogenous SLIT ligands decrease steroidogenesis in Leydig cells *in vitro*.** *Expression of Star, Cyp11a1 and Cyp17a1 determined by RT-qPCR* ***(A)*** *in MA10 cells and* ***(B)*** *in MLTC1 cells treated for 4 and 8 hours, respectively, with vehicle or 10 ug/ml exogenous SLIT1, -2 or -3. n=3 samples per group. Expression of each transcript was normalized to the housekeeping gene Rplp0. Data are means ± sem ; statistical analysis (Student’s T-test) : * p<0,05 ; ** p<0,01 ; *** p<0,001.*

*
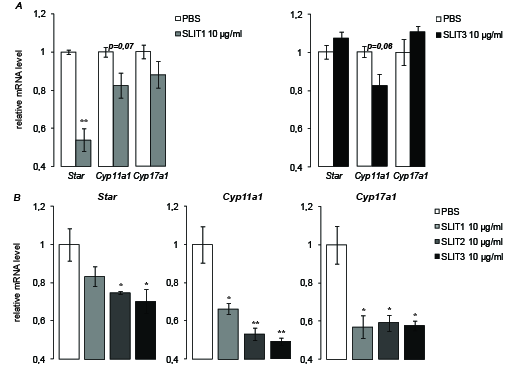
*

**Additional file 1: Figure S3. Validation of the efficiency of Leydig cell isolation.** *Expression of Star, Fshr and Dmc1 determined by RT-qPCR in whole testis and Leydig cells (n=4-6 per group). The relative levels are represented as delta-Ct versus Actb. Data are means ± sem ; statistical analysis (Student’s T-test) : * p<0,05 ; ** p<0,01 ; *** p<0,001.*

**
